# Eventration of right diaphragm with an intrathoracic ectopic kidney: A case report

**DOI:** 10.1016/j.amsu.2020.07.034

**Published:** 2020-07-27

**Authors:** Gunadi, Naisya Balela, Marcellus, Aditya Rifqi Fauzi, Andi Dwihantoro

**Affiliations:** Pediatric Surgery Division, Faculty of Medicine, Public Health and Nursing, Universitas Gadjah Mada/Dr. Sardjito Hospital, Yogyakarta, Indonesia

**Keywords:** Congenital diaphragmatic eventration, Intrathoracic ectopic kidney, Very rare disorder, Personalized surgical repair

## Abstract

**Introduction:**

Congenital diaphragmatic eventration is characterized by the elevation of the diaphragm, causing a protrusion of the intraabdominal viscera into the affected hemithorax and resulting in respiratory distress. Diaphragmatic eventration with an intrathoracic ectopic kidney is a very rare disorder with the incidence of 0.25% of all ectopias.

**Presentation of case:**

A 16-day-old male presented with chief complaint of respiratory distress. His plain chest X-ray showed intestinal gases in the right diaphragm and elevation of the right diaphragm. Intraoperative findings revealed elevation of the right diaphragmatic dome and visceral displacement, including the ileum, transverse colon, and right lobe of the liver. Subsequently, hemidiaphragm plication was conducted. Two weeks after surgery, the patient suffered from respiratory distress again. Computed tomography (CT) scanning revealed right diaphragmatic elevation and an ectopic kidney inside the right hemithorax. During the second operation, there were no longer elevation of the right diaphragmatic dome nor any other organ displacement. Moreover, we decided to let the intrathoracic kidney remain in place. The outcome was good during the postoperative period and six months after surgery.

**Discussion:**

Eventration of diaphragm with an intrathoracic ectopic kidney should be considered as a differential diagnosis in neonate patients with respiratory distress accompanied by a thoracic mass.

**Conclusion:**

Congenital diaphragmatic eventration with an intrathoracic ectopic kidney is a very rare disorder, requiring a personalized surgical repair to achieve a good outcome. CT scanning may help confirm the diagnosis, particularly to define the dome elevation and the intrathoracic organ precisely.

## Highlights

• CT-scan should be performed to establish the diagnosis of eventration of diaphragm and to find an incidental, very rare intrathoracic finding, such as an ectopic kidney.
• Diaphragmatic eventration with an intrathoracic ectopic kidney is a very rare disorder, requiring personalized surgical repair to achieve a good outcome.
• Ectopic kidney might be left intrathoracic without any complications and not require any surgical treatment.

## 1 Introduction

Diaphragmatic eventration is an abnormal elevation of the diaphragm, that may originate from congenital or acquired defects [1]. Congenital diaphragmatic eventration is caused by developmental abnormalities, characterized by diaphragmatic muscular aplasia, involving a protrusion of the intraabdominal viscera into the affected hemithorax and resulting in respiratory distress [1], with incidence of <0.05% [2]. Ectopic kidney is a rare disorder with the incidence of 0.02–0.2% in the general population, while intrathoracic ectopic kidney is the rarest form of ectopic kidneys with the incidence of <5% of all ectopias [3]. Most cases are asymptomatic and discovered unintentionally while undergoing chest radiography [4]. Diaphragmatic eventration with an ectopic intrathoracic kidney is a very rare disorder with the incidence of 0.25% of all ectopias, requiring a personalized surgical repair to achieve a good outcome [3]. Moreover, it presents both clinical and diagnostic challenges due to variations in defect location, sex, and age at diagnosis [3].

In this report, we presented a very rare case of eventration of the right diaphragm with an intrathoracic ectopic kidney. This case report has been reported in line with the SCARE 2018 criteria [5].

## 2 Presentation of case

**Fig. 1:**
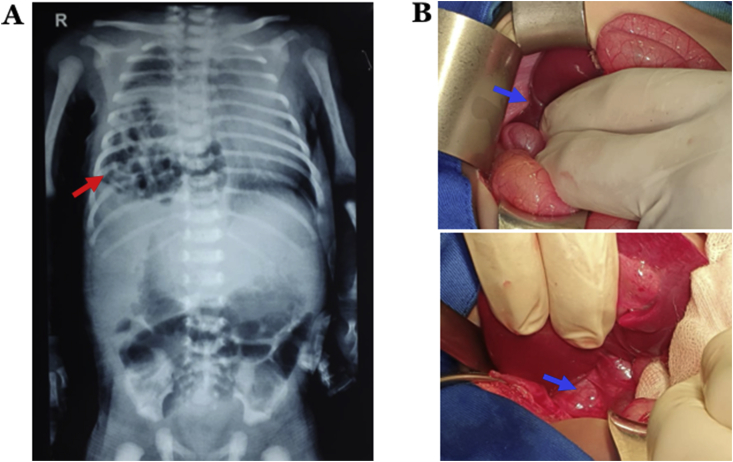
A) Chest X-ray showed intestinal gases on the right diaphragm and elevation of the right diaphragm. B) Intraoperative findings revealed elevation of the right diaphragmatic dome and visceral displacement, including the ileum, transverse colon, and right lobe of liver; and no diaphragmatic rupture was found.

A 16-day-old-male presented with chief complaint of respiratory distress when brought by his parents to the emergency unit of Dr. Sardjito Hospital, Yogyakarta, Indonesia. His vital signs were heart rate of 152 beats/minutes, respiratory rates of 66 times/minutes, temperature of 36.5 °C and oxygen saturation of 98% on oxygen support by nasal cannula of 2 L/minutes. Chest examination showed retraction on both sides of the thorax, decreased breath sounds which resounded with crepitant rales on the right hemithorax, and bowel sounds were heard on the right hemithorax. No indications of scaphoid abdomen nor other signs were noted. In addition, there was no similar disorder in his family history. He was then transferred into the neonatal intensive care unit (NICU). Laboratory findings were hemoglobin of 14.4 g/dL, white blood cell counts of 10,640/mm3, platelet counts of 442,000/mm3, C-reactive protein of <5 mg/dL, sodium of 139 mmol/L and potassium of 6.18 mmol/L. Blood gas analyses were pH of 7.307, PCO_2_ of 41.7 mmHg, PO_2_ of 45 mmHg, HCO3 20.8 mmol/L, BE of −5 mmol/L, and SO_2_ of 77%. His plain chest X-ray showed intestinal gases on the right diaphragm and elevation of the right diaphragm, which were consistent with diaphragmatic eventration (Fig. 1A). His provisional diagnosis was congenital diaphragmatic eventration. The pediatric surgeon decided to perform open repair per laparotomy according to the plain chest X-ray findings since the respiratory distress was getting worse. Intraoperative findings revealed elevation of the right diaphragmatic dome and visceral displacement, including the ileum, transverse colon, and right lobe of the liver. Furthermore, because no diaphragmatic rupture was found, the case was then determined as diaphragmatic eventration (Fig. 1B). Subsequently, hemidiaphragm plication was conducted using prolene 0. Improvement of respiratory symptoms was noted.

**Fig. 2:**
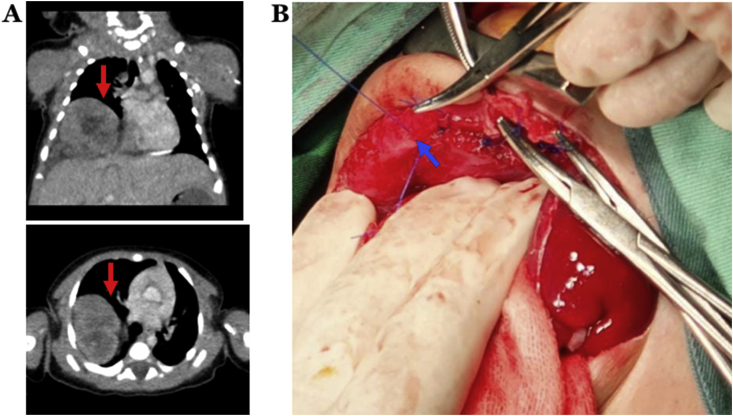
A) Computed tomography (CT) scanning indicated right diaphragmatic elevation and kidney inside the right hemithorax. B) Intraoperative findings displayed neither elevation of right diaphragmatic dome nor any other organ displacement.

Two weeks after surgery, the patient suffered from respiratory distress again. Then, computed tomography (CT) scanning was conducted and revealed right diaphragmatic elevation and an ectopic kidney inside the right hemithorax (Fig. 2A). At 20 days after the first surgery, we performed the second surgery. During the operation, there was no longer elevation of the right diaphragmatic dome nor any other organ displacement (Fig. 2B). Moreover, we decided to let the intrathoracic kidney remain in place. Meanwhile, the patient recovered uneventfully and was extubated at three days after surgery. The patient was discharged from the NICU at eight days after the second surgery. Total length of stay of patient was 30 days in the NICU and no any in-hospital complications were found.

**Fig. 3:**
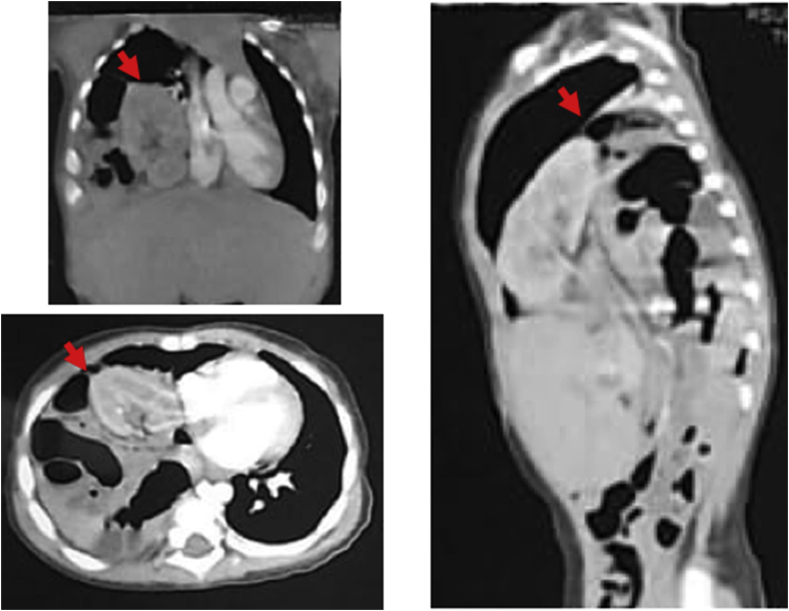
CT scanning exhibited the elevation of right diaphragmatic dome, with the 7th segment of the liver and transverse colon adhered to the right diaphragmatic dome.

At 5 months after the second surgery, the patient's complaints were relieved but sometimes a mild cough still occurred. CT-scan was performed and again showed the elevation of the right diaphragmatic dome, with segment the 7th of the liver and transverse colon adhered to the right diaphragmatic dome (Fig. 3). Unfortunately, the parents refused further investigation because they considered the patient is still in good health, however, they agreed that their son will be followed up regularly in our hospital. Moreover, his developmental milestones were as expected for his age.

## 3 Discussion

Here, we presented a very rare case of right diaphragmatic eventration with an intrathoracic ectopic kidney. Eventration of diaphragm should be considered as a differential diagnosis in neonate patients with respiratory distress [1]. Interestingly, in our case, besides right diaphragmatic eventration, there is an intrathoracic ectopic kidney.

Currently, the exact mechanism of ectopic kidney is unclear, particularly for the kidney that is intrathoracic. It is estimated that the mechanism for this condition is that the development phase of the adrenal glands and liver changes the kidney position, and the kidneys develop secondary to the nephrogenic cord [6].

The association between an intrathoracic ectopic kidney and the diaphragmatic eventration varies among reports. In most cases, the ectopic kidney is diagnosed incidentally and remains asymptomatic. Possible causes of this case are muscle fiber insufficiency due to paralysis, aplasia, or atrophy in the prenatal period; this condition is a very rare. Also, it has been reported that the ectopic kidney could also be found with multiple system anomalies [7].

Furthermore, intrathoracic ectopic kidney should be considered as one of the differential diagnoses of the thoracic mass. Investigations such as CT scans can help confirm the diagnosis. CT scanning can be utilized to define the dome elevation and the intrathoracic organ precisely [3], such as an ectopic kidney as in our case. In our patient, the intrathoracic ectopic kidney did not require further medical nor surgical treatment as previously reported [8].

## 4 Conclusions

Congenital diaphragmatic eventration with an intrathoracic ectopic kidney is a very rare disorder, requiring a personalized surgical repair to achieve a good outcome. CT scanning may help confirm the diagnosis, particularly to define the dome elevation and the intrathoracic organ precisely.

## Consent

Written informed consent was obtained from the patients’ parents for publication of this case report and accompanying images. A copy of the written consent is available for review by the Editor-in-Chief of this journal on request.

## Funding

The article processing charge of this article was funded by the Faculty of Medicine, Public Health and Nursing, Universitas Gadjah Mada.

## Ethical approval

The informed consent form was declared that patient data or samples will be used for educational or research purposes. Our institutional review board also do not provide an ethical approval in the form of case report.

## Author contribution

Gunadi conceived the study and write the final draft. Aditya Rifqi Fauzi write the original draft. Naisya Balela and Marcellus curated the data. Andi Dwihantoro supervised the study. Gunadi, Naisya Balela, Marcellus, Aditya Rifqi Fauzi, and Andi Dwihantoro facilitated all project-related tasks.

## Guarantor

Gunadi.

## Declaration of competing interest

No potential conflict of interest relevant to this article was reported.
